# Laparoscopic splenectomy using conventional instruments

**DOI:** 10.4103/0972-9941.16529

**Published:** 2005-06

**Authors:** A. N. Dalvi, P. M. Thapar, A. A. Deshpande, S. A. Rege, R. Y. Prabhu, A. N. Supe, R. S. Kamble

**Affiliations:** Department of General Surgery, King Edward VII Memorial Hospital and Seth G.S. Medical College, Mumbai, India; * Department of Surgical Gastroenterology, King Edward VII Memorial Hospital and Seth G.S. Medical College, Mumbai, India

**Keywords:** Laparoscopy, minimal access surgery, splenectomy

## Abstract

**Introduction:**

Laparoscopic splenectomy (LS) is an accepted procedure for elective splenectomy. Advancement in technology has extended the possibility of LS in massive splenomegaly [Choy et al., J Laparoendosc Adv Surg Tech A 14(4), 197–200 (2004)], trauma [Ren et al., Surg Endosc 15(3), 324 (2001); Mostafa et al., Surg Laparosc Endosc Percutan Tech 12(4), 283–286 (2002)], and cirrhosis with portal hypertension [Hashizume et al., Hepatogastroenterology 49(45), 847–852 (2002)]. In a developing country, these advanced gadgets may not be always available. We performed LS using conventional and reusable instruments in a public teaching the hospital without the use of the advanced technology. The technique of LS and the outcome in these patients is reported.

**Materials and Methods:**

Patients undergoing LS for various hematological disorders from 1998 to 2004 were included. Electrocoagulation, clips, and intracorporeal knotting were the techniques used for tackling short-gastric vessels and splenic pedicle. Specimen was delivered through a Pfannensteil incision.

**Results:**

A total of 26 patients underwent LS. Twenty-two (85%) of patients had spleen size more than 500 g (average weight being 942.55 g). Mean operative time was 214 min (45–390 min). The conversion rate was 11.5% (*n* = 3). Average duration of stay was 5.65 days (3–30 days). Accessory spleen was detected and successfully removed in two patients. One patient developed subphrenic abscess. There was no mortality. There was no recurrence of hematological disease.

**Conclusion:**

Laparoscopic splenectomy using conventional equipment and instruments is safe and effective. Advanced technology has a definite advantage but is not a deterrent to the practice of LS.

## INTRODUCTION

The first successful laparoscopic splenectomy (LS) was reported by Delaitre and Maignien.[5] This procedure over the last decade has been accepted as the treatment of choice for patients with idiopathic thrombocytopenic purpura (ITP) who fail to respond to medical treatment.[6] Technological developments like harmonic scalpel, endoscopic vascular staplers, ligasure, and hand port have aided the surgeons in removing large spleens and spleens in portal hypertension.[4][7] In a developing country due to the cost factor; not all the hospital set ups have access to this technology. Moreover the size of spleen and the disease spectrum varies from that in western countries. Starting in 1998, we have performed 26 laparoscopic splenectomies using reusable conventional instrumentation, electrocautery, clips, and intracorporeal knotting with success. We conclude that while advanced gadgets have their own importance, nonavailability should not deter a laparoscopic surgeon from performing LS.

## MATERIAL AND METHODS

Since 1998, all patients in whom elective splenectomy was indicated were included in this study.

Exclusion criteria for laparoscopic approach were:

- High-anesthesia risk due to cardiorespiratory and allied conditions;
- Portal hypertension;
- Trauma.

The patients were primarily evaluated by hematologist and referred for splenectomy after complete workup. Blood investigations included a complete hemogram, platelet count, smear examination, hemolytic studies, and coagulation profile. Blood sugars, liver and renal profiles were done as a part of overall fitness for anesthesia and for detection of coexisting diseases. Patients with splenomegaly underwent esophagogastroduodenoscopy to rule out varices before surgery. Bone marrow examination was done in select cases (hypersplenism, lymphoma, etc.).

Ultrasonography was done for splenic size and for detection of coexisting gallstones. Contrast enhanced computerized tomography (CECT) was done for evaluation of vascular anatomy of spleen, presence of accessory spleens, and presence of enlarged lymph nodes in suspected malignancy. Celiac angiography was done in one patient for suspected vascular malformation in the spleen (hamartoma).

Antipneumococcal vaccine was given 3 weeks prior to surgery. Perioperative broad-spectrum antibiotics were given to all patients. Patients on oral steroids were given intravenous steroids perioperatively. Patients were admitted 1 day prior to surgery. Bowel was prepared using polyethylene glycol. Blood and blood products, e.g., platelets in ITP, hypersplenism were kept ready as per need. Informed consent was taken for laparoscopic removal of spleen after explaining the risks, complications, and need for conversion.

### Technique of surgery

All procedures were performed under general anesthesia. Nasogastric tube and Foley catheter were passed. The patient was given 60° right lateral position and a 10–15° head high position that helped the spleen to hang from its posterior attachments and kept the stomach away due to effect of gravity. The surgeon and camera assistant stood on patients right side, second assistant on patient's left side. Open technique for first trocar insertion was used. Carbon dioxide was used for insufflations with intra-abdominal pressure set at 12–14 mmHg. The patient's abdominal contour, subcostal angle, and the probable site of the vascular pedicle of the spleen determined the port placements. For the telescope (30°), 10 mm port was made usually along the line joining the umbilicus to left midsubcostal line.

Two 10 mm working ports were placed on either side of the telescope to maintain coaxial vision. A 5-mm fourth port was placed in the left anterior or midaxillary line to retract the spleen and use of suction when necessary.

Additional fifth 5-mm port at the xiphisternum was required in seven cases to retract the left lobe of liver. The gastrocolic ligament was divided by short bursts of coagulation current that enabled sealing of the short-gastric vessels without jeopardizing the stomach. If the short-gastric vessels were greater than 4 mm in diameter, bipolar current or clips (Ligaclips, Ethicon Inc were used. A thorough examination was carried out for presence of accessory spleens or splenenculi at this stage.

The splenic artery was identified and dissected at the superior border of the pancreas and doubly ligated by intracorporeal knotting with 2-0 silk, clipped and cut.

The splenocolic ligament was next dissected bringing down the splenic flexure of the colon. The position of the patient and effect of gravity kept the colon away from the operative field.

Hilar dissection was then carried out from the lower pole upwards staying close to the hilum avoiding the pancreatic tail. The main splenic vein was identified, dissected and ligated doubly with 2-0 silk and cut. The smaller pedicles at the poles, if encountered were clipped and cut.

The lienorenal ligament and diaphragmatic attachments were cut using monopolar current making the spleen free of all its attachments.

Accessory spleens if located were dissected and their vascular pedicle clipped.

The spleen was delivered out through a 5–8 cm pfannensteil incision. Hemostasis was confirmed. A drainage tube was placed in all but one patient through the left lateral port. Ports were closed with 1/0 vicryl.

In three patients with coexisting gall stones, patient were turned to supine position after splenectomy and laparoscopic cholecystectomy was completed.

Nasogastric tube was removed on day 1 postoperation and patients were started on oral feeds. Complete hemogram was done and platelet count was checked in all patients on day 1. Steroids were gradually tapered in patients on preoperative steroids.

### Method

All the patients were analyzed with respect to indications, weight of spleen, duration of surgery, conversion to open surgery, duration of in-hospital stay, morbidity, and mortality.

## RESULTS

A total of 26 patients underwent LS in our center from 1998 to 2004, by a single team of surgeons. Age of patients ranged from 14 to 60 years with a mean of 28 years. There were 17 males and nine females. Three of these patients also underwent laparoscopic cholecystectomy for gallstones.

Indications for LS are detailed in Table 1. Tropical splenomegaly (*n* = 8) with symptomatic hypersplenism was the commonest indication.

**Table 1:** Indications for laparoscopic splenectomy [total number of patients (1998–2004) – 26]

Indications	No. of patients
Tropical splenomegaly	8
Hereditary spherocytosis	6 laparoscopic cholecystectomy (*n* = 3)
Autoimmune hemolytic anemia	4
Idiopathic thrombocytopenia purpura	4
Thalasemia major	2
Non-Hodgkin lymphoma	1 use of hand (glove-in-glove technique)
Splenic hamartoma	1

**Figure 1:**
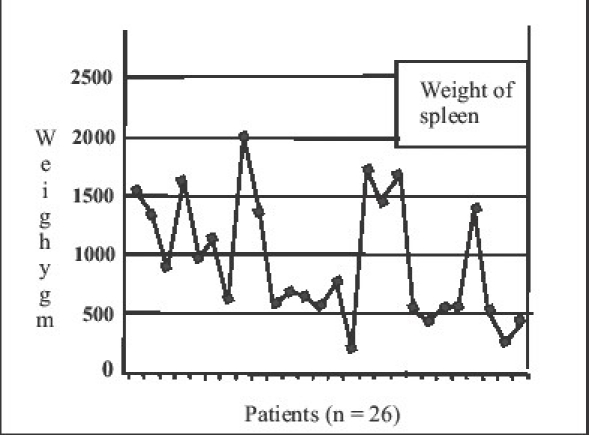
Weight of spleen

Spleen weight was more than 500 g in 85% of patients (*n* = 22) and more than 1000 g in 38.46% of patients (*n* = 10). The average weight of the spleens removed was 942.5 g (range 200–2000 g). The graph [Figure 1] depicts the weight chart of the spleens removed. One patient having massive spleen due to suspected non-Hodgkin Lymphoma required the use of hand assist where we used ‘glove-in-glove’ technology.

**Figure 2:**
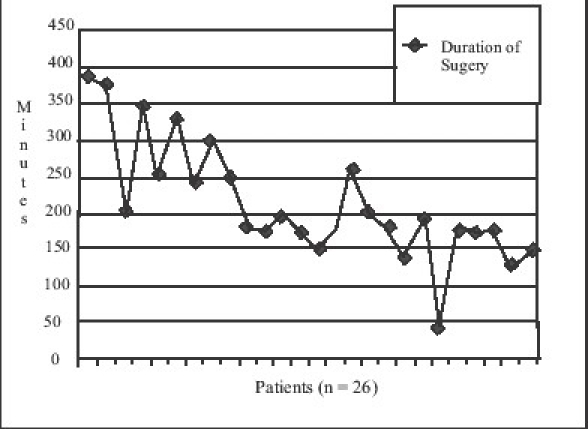
Duration of Surgery

The average duration of surgery was 214 min (range 45–390 min). The graph [Figure 2] depicts the duration of surgery in each case.

Average blood loss in successful laparoscopic cases was 170 cm^3^. In two patients, who were converted, the blood loss was 800 and 1200 cm^3^, respectively.

A 4-cm transverse subumbilical incision was taken to deliver the spleen in the first case. Spleen was delivered through a left subcostal incision in three patients who were converted to open surgery. Five- to eight-centimeter pfannensteil incisions were taken for delivery of spleen in the remaining 22 patients.

Three patients required conversion to open surgery (11.5%). In two of these patients, bleeding from upper polar vessels was the cause of conversion. These were initial cases (Cases 2 and 3). In one patient, the conversion was related to malfunction of CO_2_ insufflator (Case 7).

The immediate postoperative period was uneventful in all but two patients. One patient of tropical splenomgaly developed sepsis syndrome that responded to higher antibiotics and patient was discharged on seventh day. One patient developed a left subphrenic abscess, which was drained by the posterior extraperitoneal approach as described by Nather and Ocshner.[8] In this patient, the pancreatic tail was embedded in the splenic hilum leading to difficult dissection in that area. Excluding this patient, who had to be hospitalized for 30 days, the average postoperative the hospital stay was 4.68 days (range 3–7 days). There was no mortality.

Accessory spleens were encountered and removed in two patients. In the first, it was present around the hilum along the inferior border of the pancreas. The second patient had multiple spleninculi in the hilum and was delivered as part of the specimen.

The longest follow up is 720 months and shortest is 5 months. No patient has developed recurrence of the hematologic disease.

## DISCUSSION

Since Philip Mouret described the procedure of laparoscopic cholecystectomy, improvement in laparoscopic skills and boom in technology revolutionized the world of general surgeons into the world of laparoscopic surgeons. Laparoscopic splenectomy was first documented by Delaitre and Maignien in 1991.[5] Splenomegaly was initially considered to be a contraindication for laparoscopic intervention.[9] Small size of spleen in ITP therefore became the commonest indication for LS.[6][10] Introduction of harmonic scalpel,[11] endovascular stapler,[12] and hand port[13] allowed removal of large spleens by laparoscopic technique. Recent reports suggest a trend towards the use of hand port (assisted surgery) in the treatment of massive splenomegaly requiring splenectomy.[13][14] Having the hand inside of the abdomen provides the all important touch perception, helps retraction and dissection, control of bleed in necessity, and retrieval of the specimen.[15] Costs of these devices prevented us an access to them and hence not used in our public funded the hospital setting.

The total number of patients operated upon is 26. In spite of acceptability of LS in the Western world, there seems to be aversion to referrals for splenectomy (open or LS) from the hematology group in our country. This experience by the authors is similar to that reported in cases of gastroesophageal reflux disease (GERD) by Balsara et al.[16] and Sarani et al.[17] Further, the patient is always afraid to undergo surgical intervention when alternative option is made available.

Tropical splenomegaly or idiopathic splenomegaly is a common condition encountered in Indian setting as against ITP in the west. Symptomatic hypersplenism or effects of large spleen becomes an indication for splenectomy in these patients. Hereditary spherocytosis and autoimmune hemolytic anemia, more common in our setting also have moderately enlarged spleens. Palanivelu have reported three-fourth of their series of splenectomies being performed for large spleens.[18] In our series, 22 patients had spleens weighing more than 500 g; ten patients had spleen more than 1000 g indicating that splenomegaly is not a contraindication for LS with conventional instruments.

**Figure 3:**
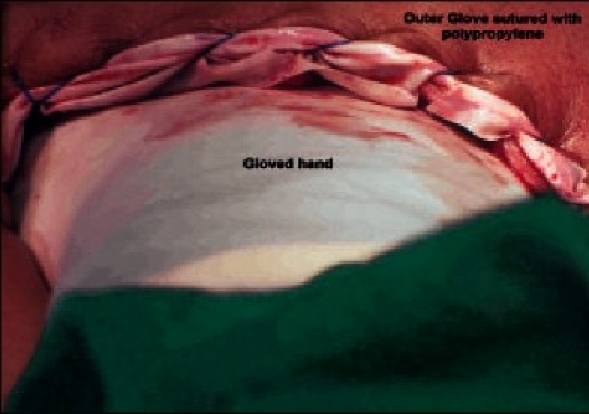
Photograph showing “glove in glove” technique

Laparoscopic splenectomy was successfully performed in 22 patients. In one patient of massive splenomegaly due to non-Hodgkin Lymphoma (weight 1720 g), we used ‘glove-in-glove technology’ as a hand port to circumvent the cost. A (GammexÒ Ansell Inc.) 71/2 size glove was sutured with 3/0 polypropylene continuous sutures to the sheath of the 4-cm incision to prevent gas leak. This particular glove was used as it had greater elasticity to reach distant areas without the risk of tears. The surgeon/assistant's gloved hand was introduced through this glove to handle and manipulate the spleen [Figure 3]. This procedure was a success under the nomenclature of ‘glove-in-glove hand-assisted’ LS. Walsh et al. and Cavaliere et al. have also reported successful outcome with hand-assisted LS (HALS) in malignant	hematological diseases of spleen.[19][20]

The main objective of port placements should be to tackle the splenic pedicle easily. The abdomen of our patient is usually smaller with more acute subcostal angle when compared to from west. We feel that the port placement be planned after examining the inflated abdomen and the splenic size. An enlarged spleen forces the surgeon to be away from the ‘left subcostal’ port placement to avoid instrument congestion as against that described in western literature.

All the positions – supine, lateral, and angled have been described. The initial literature described supine position for LS.[21] Lateral or angled positions are preferred today.

Two approaches – the ‘anterolateral approach’ and the posterior or ‘posterolateral approach,’ are described for LS. The posterior (‘posterolateral detached spleen’) technique was described by Park et al.[22] Surgeons who use endostaplers for pedicle transection prefer lateral position and tend to use posterolateral approach. In this approach, the spleen is retracted medially after dividing the splenophrenic and splenorenal ligaments. The pancreatic tail is visualized and so is the splenic pedicle. The approach is reported to have a lesser incidence of pancreatic complications and bleeding[23] but requires experience with retroperitoneal laparoscopic dissection; technique unfamiliar to an average laparoscopic surgeon with basic training in open splenectomy by transabdominal technique. Further, the use of endoscopic vascular stapler for the splenic pedicle appears to be mandatory in this technique.

We practice the ‘general surgeon familiar’ anterolateral approach (also known as the ‘hanging spleen technique’) using the right lateral position with 60–80° tilt. This helps in a ‘general surgeon perception’ of splenic anatomy as in open surgery and is easier to manage in case of conversion to laparotomy.

Use of harmonic scalpel has become a routine in LS. We find that careful use of small intermittent bursts of monopolar coagulation that does not allow lateral spread of current is effective for coagulation of short-gastric vessels. We are of the opinion that the gastrocolic ligament gets elongated as the spleen enlarges, thereby helping in safe use of electrocautery. Short-gastrosplenic ligament was encountered in four cases (ITP), where bipolar coagulation or clips were used. Effect of position and gravity after the dissection of the gastro colic ligament retracts the viscera and exposes the pancreatic tail and the splenic pedicle. This gives direct access to the splenic artery and vein. Arterial ligation changes the color of the spleen from brown to blue/violet and is a useful indicator for the extent of devascularization of the spleen and a clue to an additional arterial supply. Early ligation is reported to reduce the size and decrease blood loss during surgery.[11] The proximal side of the splenic vein is ligated first. This prevents engorgement of the vein and eases ligation on the distal side. The splenorenal ligament is incised last in contrast to the posterior approach, thus serving the purpose of hanging spleen technique.

The literature review reveals that the spleen is delivered by use of commercially available puncture resistant bags. The bags are delivered out through one of the ports and the spleen morcellated before removal.[11] The cost of these bags prevented us from their use. Some surgeons apparently use cheaper autoclaved polyethylene bags for retrieval but there is a fear of unwanted spillage of hematological splenic tissue into the peritoneal cavity in case of puncture in these bags. Most of the spleens in our series were large (average weight 942.5 g) and it is cumbersome to place large spleens in retrieval bags.[15] The length of the pfannenstiel incision in our series was 5–8 cm that is similar to that described by Tagarona et al.[15] in his technique of HALS for large spleens. Also, a limited pfannenstiel incision is reported to be less painful than conventional subcostal incision.[11]

In our series accessory spleens were encountered intraoperatively in two patients. Morris et al. reported 0–12% incidence of identification of accessory spleen during LS, and successful laparoscopic removal of missed accessory spleen.[24] Though Katkhouda et al. in a study of 103 LS suggested limited use of imaging techniques in prediction of accessory spleens,[25] Napoli et al. have reported accurate assessment of splenic pathology and accessory spleens by using multidetector row CECT angiography.[26]

The average time taken for the procedure has been 214 min in our series. This has declined with experience, not significantly however, and can certainly be attributed to use of conventional instruments. Rosen et al. in their series of 147 LS with the use of harmonic scalpel, have reported average time of 134 min for small size spleen, and 170 min for massive splenomegaly.[10] The operative time is important in the west as it automatically escalates the cost of the procedure. Though the use of advanced instruments decreases the operative time, their use increases the overall cost of splenectomy (US $1664 for open against US $2064 for LS).[27] In our set up, the treatment to patient is free and hence the operative time is not a major determinant of the cost factor as in the west or private Institutional set ups. The overall mean duration of stay in our study was 5.65 days (range being 3–30 days), higher than that reported in literature. Hamamci et al.[27] have documented reduction in the hospital cost of LS in developing countries compared to open splenectomy due to less morbidity and shorter the hospital stay and the same was our experience.

The complications of LS as a procedure are the same as that of open splenectomy ranging from intraoperative bleeding, postsplenectomy sepsis, wound/port site infection and pancreatic injury. Terrosu et al. reported higher incidence of conversion, bleeding, and morbidity in spleens greater than 2000 g.[28] One patient of tropical splenomegaly (weight 1440 g) developed frank acute pancreatitis in the immediate postoperative period and later developed abscess in the left subdiaphragmatic space that required drainage On retrospective review, it was seen that the pancreatic injury had occurred during dissection of the vein as the pancreatic tail was embedded within the splenic hilum. The patient stayed in the hospital for 30 days. The literature does report a 6% incidence of benign pancreatic reaction[29][30] and a slightly higher risk of pancreatic injury (9.5%)[31] in LS.

The impact and efficacy of advanced equipment and instrumentation in Laparoscopic surgery seem to have laid foundation for advanced procedures in surgery including LS. Our series shows that LS is possible with conventional equipment and instrumentation. Complication rate and long-term outcome is not affected.

## CONCLUSION

Laparoscopic splenectomy using conventional equipment and instruments is safe and effective. Advanced technology has a definite advantage but is not a deterrent to the practice of LS. Innovations like ‘glove-in-glove’ technology can help. The average time taken is more with conventional instruments, but the overall cost is lesser specially when the operative time is not a determinant of the cost.

Laparoscopic splenectomy though has a definite learning curve, is not difficult if one has experience in open splenectomy. The procedure of LS has the same benefits of minimal invasive surgical techniques as against open surgery and should become the surgeon's procedure of choice soon even if advanced technology is unavailable.
